# Relationship between Speed of Response Inhibition and Ability to Suppress a Step in Midlife and Older Adults

**DOI:** 10.3390/brainsci11050643

**Published:** 2021-05-15

**Authors:** Derek England, Kathy L. Ruddy, Christopher J. Dakin, Sarah E. Schwartz, Blake Butler, David A. E. Bolton

**Affiliations:** 1 Department of Kinesiology and Health Science, Utah State University, Logan, UT 84322, USA; derekengland@aggiemail.usu.edu (D.E.); chris.dakin@usu.edu (C.J.D.); 2 School of Psychology and Institute of Neuroscience, Trinity College Dublin, Dublin D02 PN40, Ireland; ruddykl@tcd.ie; 3 Sorenson Legacy Foundation Center for Clinical Excellence, Utah State University, Logan, UT 84322, USA; 4 Department of Psychology, Utah State University, Logan, UT 84322, USA; sarah.schwartz@usu.edu; 5 Department of Sport and Exercise Science, University of Northern Colorado, Greeley, CO 80639, USA; butl4276@bears.unco.edu

**Keywords:** response inhibition, reactive balance, aging, executive function, stepping

## Abstract

In young adults, performance on a test of response inhibition was recently found to be correlated with performance on a reactive balance test where automated stepping responses must occasionally be inhibited. The present study aimed to determine whether this relationship holds true in older adults, wherein response inhibition is typically deficient and the control of postural equilibrium presents a greater challenge. Ten participants (50+ years of age) completed a seated cognitive test (stop signal task) followed by a reactive balance test. Reactive balance was assessed using a modified lean-and-release system where participants were required to step to regain balance following perturbation, or suppress a step if an obstacle was present. The stop signal task is a standardized cognitive test that provides a measure of the speed of response inhibition called the Stop Signal Reaction Time (SSRT). Muscle responses in the legs were compared between conditions where a step was allowed or blocked to quantify response inhibition of the step. The SSRT was significantly related to leg muscle suppression during balance recovery in the stance leg. Thus, participants that were better at inhibiting their responses in the stop signal task were also better at inhibiting an unwanted leg response in favor of grasping a supportive handle. The relationship between a seated cognitive test using finger responses and leg muscle suppression when a step was blocked indicates a context-independent, generalized capacity for response inhibition. This suggests that a simple cognitive test such as the stop signal task could be used clinically to predict an individual’s capacity for adapting balance reactions and fall risk. The present results provide support for future studies, with larger samples, to verify this relationship between stop signal reaction time and leg response during balance recovery.

## 1. Introduction

Many of our actions are highly automated and can be accomplished with minimal cognitive effort. However, as behavioral demands deviate from what is instinctual, higher brain resources are necessary [1]. In the case of balance control, stereotyped, whole-body postural responses are rapidly initiated via subcortical networks to resist a loss of balance [2,3,4]. While essential, there are many situations in daily life that call for adaptation of reflexive action. A prerequisite for behavioral flexibility is the ability to prevent an automatic, yet undesired action, known as response inhibition. This ability to stop or prevent action is rarely considered in relation to how we avoid a fall, but mounting evidence indicates that executive function is related to fall prevalence, which includes inhibitory control [5,6,7,8,9,10,11]. For example, Mirelman, in 2012, conducted a prospective, longitudinal study to examine the relationship between multiple cognitive abilities and falls in >200 community-dwelling seniors. The authors revealed that executive function predicted falls over the 5 years following cognitive assessment. The executive function index in their study included tests that emphasized some form of response inhibition (e.g., Stroop and Go/No-go tests) suggesting that inhibitory control may be important in fall prevention. This premise has been supported by other studies [7,11] and in other settings where researchers examined the relationship between executive function and falls in a neurorehabilitation unit over a 1-year period [10]. Collectively, these studies suggest that inhibitory control may play a role in preventing falls even when other cognitive measures fail to correlate with falls.

Detailed assessment of voluntary stepping reactions has exposed response inhibition deficits in older adults when they perform choice reactive stepping tasks [12,13]. Cohen, in 2011, showed that Stroop performance (a classic cognitive test of inhibitory control [14,15,16]) correlated with anticipatory postural adjustment errors that preceded the step [12]. That finding suggested that what underlies the prolonged time required to respond to a choice in older adults may in fact be a deficit in response inhibition. Presumably, such delays would be even more impactful in the time-pressured context of reactive balance control where a recovery step is needed to avoid a fall. Indeed, performance on a choice stepping task (especially with an inhibitory component) is predictive of falls for one year, illustrating an important link between inhibitory deficits in stepping and actual falls [17]. Furthermore, Schoene, in 2017, demonstrated that this capacity is predictive of falls even in seemingly normal community-dwelling seniors [17]. These studies have highlighted a role for response inhibition in step reactions and revealed that inhibitory deficits in older adults could lead to step errors and response delays. This underscores the enormous value to be found in understanding more about inhibitory control and its role in fall prevention. 

The neural networks responsible for response inhibition have been established in imaging research and patient populations during performance on standard cognitive tasks [18,19]. These same networks are recruited in a range of different inhibitory control tasks, using different modalities of sensory cues and types of response. While response inhibition is typically studied in seated subjects as they perform simple (usually hand) responses to imperative cues displayed on a computer screen, there is good reason to believe that these same neural networks influence whole-body balance reactions. For example, inhibition expressed through either a hand or a foot response has been shown to rely on a common prefrontal network [20]. Additionally, in our laboratory, we have recently shown evidence for global suppression in a task-irrelevant hand muscle when a balance recovery step was blocked [21]. This global suppression in a reactive balance context is consistent with past work where task-irrelevant leg muscles were suppressed during a seated hand response task [22]. Overall, such findings support the idea that common neural mechanisms underlie response inhibition across a wide range of tasks.

Recent work in our laboratory has shown that performance on a seated response inhibition task correlates with the suppression of leg muscle activity during a balance recovery step [23]. Specifically, the stop signal task—a gold standard test of response inhibition—was used to determine each participant’s internal stopping process, or Stop-Signal Reaction Time (SSRT). In the same session, we tested a participant’s ability to suppress an automatic balance recovery step when a leg block was present using a modified lean-and-release technique developed in our laboratory [24]. Our key finding was that stopping ability was preserved in individuals across these diverse tasks. This is consistent with the aforementioned weight shifting errors during a choice-step reaction step task correlating with Stroop test performance [12]. Collectively, these results point toward a common capacity for response inhibition across tasks, which includes rapid stepping. 

In our previous study of young adults [23], we focused on responses in the step leg, based on an a priori assumption that the step leg would best reflect a decision to step or not. However, subsequent analyses indicated that a similar correlation exists between SSRT and muscle response ratio in the stance limb. It is important to recognize that a rapid step involves coordination between the stance limb and the step limb [25,26]. Before a voluntary step is initiated, postural adjustments in the stance leg are necessary to permit acceptance of full body weight and compensate for the medial-lateral destabilization accompanying the step. Such coupling between the legs has been demonstrated using external forces that resist [27] or aid [28] anticipatory postural adjustments during stepping, resulting in a delay or advance of step onset, respectively. Of relevance to the present study, cognitive effects, such as inhibitory failure, tend to manifest first as anticipatory postural adjustment errors preceding voluntary steps (highlighted above, [12]; also, see [13]). While the size of anticipatory postural adjustments may be reduced in a perturbation-evoked step versus a voluntary step [29], there remains a tight coupling between step and stance [26]. In the sequence of events comprising a step, the stance leg, by necessity, reflects an earlier stage in the step decision process, preceding the actual step. Because of the importance of both step and stance legs working together to achieve the common goal of stepping forward, we presented both herein.

In the present study, we extended recent findings in young adults by determining whether SSRT also predicted the ability to suppress rapid balance recovery steps in mid-life and older adults. While falls in adults over the age of 65 have received considerable attention, there is emerging evidence that midlife adults show a steep rise in fall prevalence, and yet this cohort remains understudied with regard to risk factors for a fall [30]. Therefore, we sought, as a test of feasibility, to determine whether we could identify a relationship between SSRT and step suppression in midlife and older adults using the same methodology applied to younger adults [23]. Given some of the unique challenges in controlling balance and avoiding falls as we age, this study provides an initial opportunity to determine if this experimental approach is feasible in an older population. We hypothesized that the relationship between SSRT and step suppression would again be present. 

## 2. Materials and Methods

### 2.1. Participants

Participants were recruited from community members living in the Cache County area by means of recruitment fliers and word of mouth. Study enrollment was open to individuals between the ages of 50 and 85. Ten healthy midlife and older adults between the ages of 51 and 75 provided written informed consent prior to participation in this study (66 +/− 8 years of age; 5 Female and 5 Male). Procedures were approved by the Utah State University Institutional Review Board, conducted in accordance with the Declaration of Helsinki. 

### 2.2. Procedures

#### 2.2.1. Stop Signal Task (SST)

The SST was custom written in Matlab (Mathworks, MA, USA), adapted from Aron and Poldrack (2006) [18], and was described in detail in our earlier study with younger adults [23]. Briefly, seated participants performed a stop signal task where they were presented with a go signal on a computer monitor and instructed to respond as quickly as possible. The go signal was either a right or left facing arrow (“<” or “>”) and participants were instructed to respond as quickly as possible by pressing the appropriate button on the keyboard (i.e., press “>” with their middle finger if the arrow points right, and “<” with their index finger if the arrow points left). They were instructed to respond quickly once the go signal appeared but refrain from responding if an auditory stop signal was heard. On 25% of the trials, an auditory stop signal followed the go cue. The outcome of this test (the SSRT) estimated an individual’s capacity for stopping a response after the stop signal has been presented; a faster SSRT represented more effective stopping. The delay between the go and stop signals is referred to as the stop-signal delay (SSD), where inhibition of the response is more difficult when the inhibitory stimulus is presented after a longer time interval. The SSD was varied across trials using a staircase algorithm to identify the time point at which participants had a 50% probability of correctly inhibiting a go response after the stop tone was presented. 

The SSRT was calculated using the integration method [31]. Specifically, we calculated the RT distribution of all go trials as well as the exact proportion of failed stop trials, p(respond|signal). We then used p(respond|signal) as the percentile from which to extract the nth RT from the Go RT distribution. SSRT was then calculated as the nth RT minus mean SSD.

Participants were instructed that going quickly and stopping successfully were equally important. While the go reaction time was included in the tracking algorithm, the more relevant factors related to the SSD and the percentage of successful versus failed stops. Notably, this test emphasized response inhibition instead of overt reaction speed. Because the actual latency of the stopping process could be directly measured, SSRT was estimated using a stochastic model. Participants performed 256 trials divided across 4 blocks with ~1 min of rest between blocks. Trial duration was 2500 ms. 

#### 2.2.2. Lean-and-Release Task

**Figure 1:**
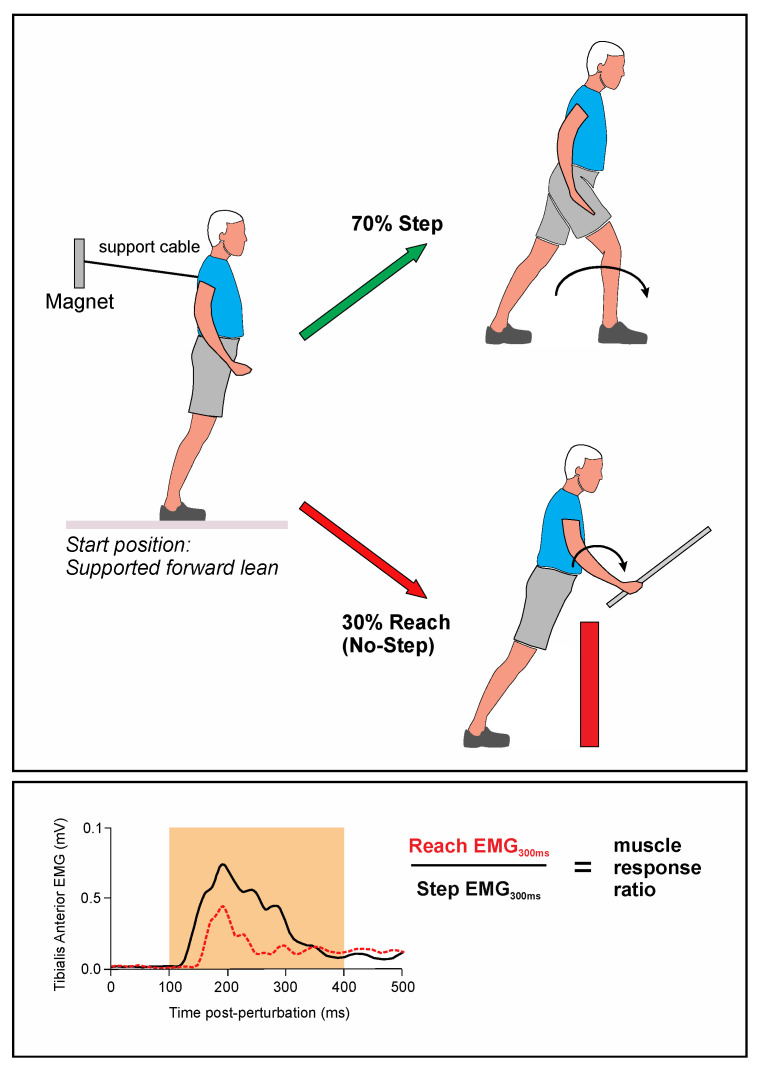
(**Top panel**): During the modified lean & release task, there are two options for recovering balance: take a step or grasp a railing (if the step is blocked). In total, 70% of trials were step trials and 30% were reach trials. In this illustration, the stance leg is the left, and step leg is the right. Below is a graphical representation of EMG activity from the tibialis anterior muscle immediately after cable release. The EMG activity is separately averaged for step trials versus reach trials (i.e., reach trials are those where a forward step is blocked and a support handle must be grasped to recover balance). (**Bottom panel**): Average rectified EMG is measured between 100 ms and 400 ms following cable release (EMG_300ms_) to calculate the muscle response ratio for each condition separately (step or reach). A low ratio means that a participant is good at suppressing a leg response during a forward step.

Following completion of the SST, reactive balance was tested using a custom-made lean-and-release system [23,24], which imposed temporally unpredictable forward perturbations. Details on this apparatus, including a video overview of the methods, were recently published [24]. Participants were placed in a harness connected by cables to the wall behind them. The experimenter instructed participants to lean as far forward as the cable allowed while keeping both feet in contact with the floor. A forward fall was imposed by magnetically releasing the supporting cable, and these postural perturbations were sufficient to force a change of support reaction consisting of either a forward step or a reach to a handrail. Following a brief familiarization session, formal testing consisted of 84 trials with rest breaks provided. For most trials (70%) no leg blocks were present, thus allowing a forward step to recover balance after cable release. Participants were instructed to step with whichever leg felt natural. This freedom to select the step leg was intended to avoid potential cognitive constraints placed on generating a rapid reaction by having to select a specific and potentially unnatural step leg. For the remaining (30%) trials, participants had an obstacle placed in front of their legs to prevent a step (see Figure 1). When this block was present, a safety handle was simultaneously uncovered to allow a compensatory reaching response. The 70:30 ratio was intended to heavily bias the stepping response, in turn forcing participants to suppress a prepotent step when the step was blocked. Note: the present study investigated the link between compensatory stepping reactions and stopping ability; thus, it was important to bias a step reaction, in the same way that a rapid button-pressing reaction is promoted in the stop signal task. The presentation of all experimental conditions was randomized by computer program. This includes the appearance/removal of a leg block and the handle-obscuring cover to ensure the correct ratio of stepping and reaching trials, as well as randomization of the viewing time described next.

The participants wore liquid crystal goggles (Translucent Technologies Inc. Toronto, ON, Canada) to occlude vision prior to the start of each trial. These goggles opened a few seconds later to reveal the specific condition. The participant was released shortly (200 ms, 400 ms or 600 ms) after the goggles opened. On a small portion of trials (14%) no perturbation was delivered to act as a catch trial, in an effort to encourage participants to act only in response to the perturbation. As a precaution, a failsafe cable was attached from the ceiling to the harness to catch the participant in the event of a fall. Throughout testing, participants were told to remain relaxed and to look at a fixation point on the ground ~1.5 m ahead. This point was adjusted as needed to ensure that the top of the leg block and the safety handle were visible in peripheral vision when the goggles opened. A Cambridge Electronic Design analog-digital recorder and Signal software (Power 1401-3A, Cambridge Electronic Design, Cambridge, UK) were used to control timing for cable release, to open/close the occlusion goggles, and to drive the servo motors in order to move the handle cover and leg block into position. 

### 2.3. Electromyography and Force Sensors

Electromyography (EMG) signals were amplified using a Delsys Bagnoli-4 amplifier (Delsys Inc., Boston, MA, USA), sampled at 5000 Hz with Signal software (Power 1401-3A, Cambridge Electronic Design, Cambridge, UK). EMG was collected from the tibialis anterior on the right (TA_R_) and left (TA_L_) legs to measure muscle activity in step and stance legs. Footswitch force sensors (B&L Engineering, Santa Ana, CA, USA) were placed inside the bottom of the shoes of each participant to detect liftoff data for step initiation. A force sensitive resistor was fixed on top of the support handle to detect contact with the handle (note: this sensor could detect contact even if the handle was covered to indicate if a grasp error occurred).

### 2.4. Analysis

EMG signals from TA_R_ and TA_L_ were band-pass filtered (10–500 Hz) and full-wave rectified. The magnitude of the EMG response was assessed as the mean, rectified EMG over a 300 ms time window starting 100 ms post-perturbation (EMG_300ms_). This time window was selected to capture the muscle response leading to a step, based on resultant liftoff times (see Results). Both step and stance limbs were analyzed using this approach. 

**Figure 2:**
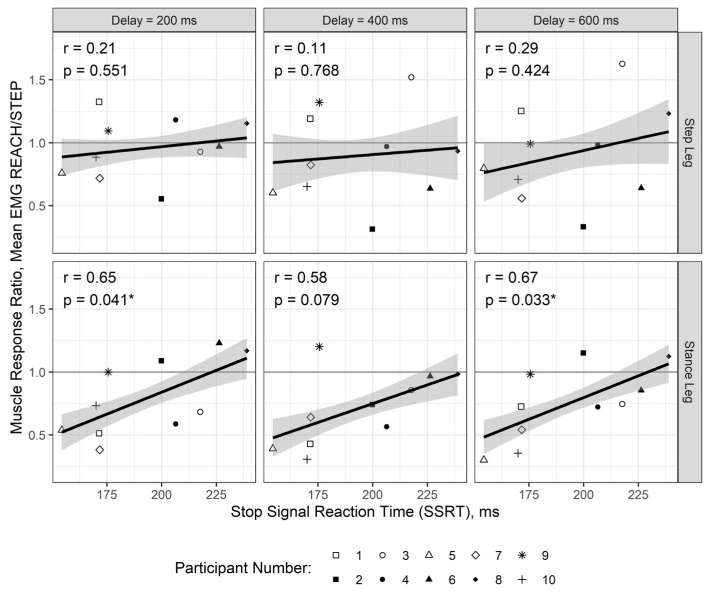
Observed aggregated muscle response ratios and stop signal reaction time by post-visual preview delay and leg. Each panel reports Pearson’s correlation among the ten observations with overlaid, independently-run linear regression and 95% confidence band. This figure is intended as a descriptive summary (n_participants_ = 10). * *p* < 0.05.

For the reactive balance test, the average EMG was assessed for each trial, and aggregated to (a) post-vision preview delay (200 ms, 400 ms, or 600 ms), and (b) condition (step or reach). Thus, each of the ten participants yielded six aggregated observations, as seen in Figure 2. The purpose was to use whichever action was afforded (step or reach) to group the EMG response in the stepping leg, not necessarily the response that actually occurred. For example, if a participant failed to suppress a step (i.e., leg block present), such trials were still classified as reach trials. The muscle response from the step leg was subsequently compared between trials where the participant should reach versus trials where they should step. A ratio was calculated to capture the difference between these two conditions by dividing the average EMG_300ms_ from the reach condition by the average EMG_300ms_ of the step condition. The assumption was that a smaller ratio represented greater success in suppressing a step. 

We used a multilevel model (MLM), also known as repeated measures (mixed effects) regression, to model the muscle response ratios and assess associations with SSRT (grand mean centered at 193.2 ms). We also investigated possible moderating effects of step vs stance leg and post-visual preview delay. Significance was assessed by comparing the nested models using Chi-squared likelihood ratio tests (LRTs), as well as Bayes factors to exclude nonsignificant terms. The parsimonious final model was decomposed using simple slopes and Nakagawa–Schielzeth marginal pseudo-R^2^ calculations. All analyses were conducted in R 3.6.2 [32]; MLM performed with the ‘lme4’ [33], ‘r2glmm’ [34] and ‘performance’ packages [35]. Post-processed data, R code, and outputs, including rationale for analyses not presented in the results (such as Bayes factors), are available in supplemental materials on the Open Science Framework (https://osf.io/2j3fe/; accessed on 19 January 2021).

## 3. Results

### 3.1. Stop Signal Task

Median GoRT was 501 ms (SEM: 13). All participants exhibited a probability of stopping between 40–60%, thus supporting the efficacy of the SSD staircase algorithm. Average SSRT was 193ms (SEM: 9; range: 154–239 ms). Average SSD was 309ms (SEM: 19). Miss rates and error rates were low (both <1%). Median RTs on failed stop trials were faster than those on Go trials for every participant, verifying the legitimacy of the horse-race model, which suggests that inhibitory and go commands are independent runners. Further, at a group level, this difference in RTs was significant (t(9) = 8.48, *p* < 0.001). In addition, there was no relationship between GoRT and SSRT (r = −0.475, *p* = 0.165). According to Verbruggen et al. [36] the aforementioned tests indicate that SSRT could be reliably estimated in our sample. 

### 3.2. Lean-and-Release Task

One of the ten participants stepped with their left leg on all occasions. The remaining nine participants stepped primarily with their right legs. The few trials in which a right stepper used a left step instead were not included in subsequent analyses. Liftoff times across all participants were on average 471 ms (SEM 34 ms). These liftoff times were used to define our analysis window for the muscle response ratio (i.e., 100–400 ms) to ensure we captured the majority of muscle activity leading to the step response. This window was applied to both the stance limb and stepping limb. Footswitch and the grasping handle force data were used to identify step and reach-to-grasp errors, respectively. Reach-to-grasp errors (i.e., grasping the handle when covered) were rare and only occurred in about 2% of the step trials. Step errors (i.e., stepping when a leg block was present and the handle was available), on the other hand, were found in 38.3% of the reach trials, but this was highly variable between participants. For example, three participants made two or fewer step errors, whereas one participant stepped on almost on every leg block trial. Importantly, no participant responded to any of the catch trials.

### 3.3. Relationship Between Muscle Activation in the Legs and Stop Signal Reaction Time 

**Figure 3:**
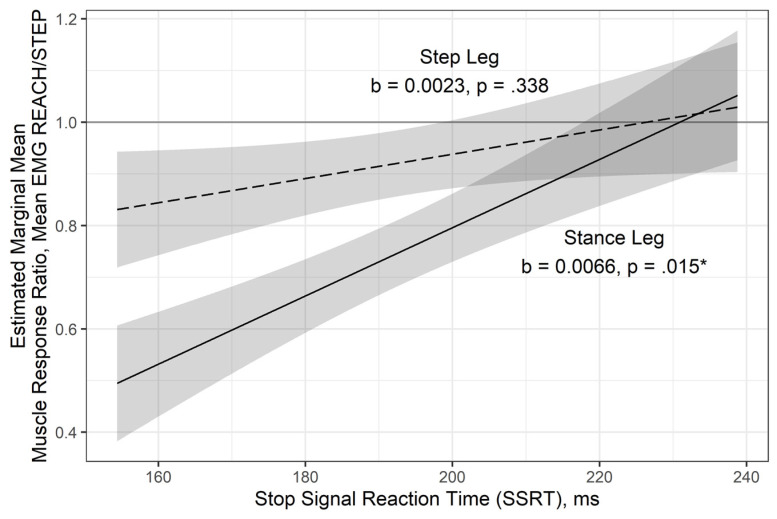
The multilevel model (MLM) and simple slopes analysis identified a relationship between the SSRT and muscle response ratio for the step and stance legs without undue aggregation (ecological fallacy) or risk of type I error rate inflation (multiple comparisons). This model displays strong evidence of a significant association between the SSRT and muscle response ratio (i.e., tendency to suppress a leg response) in the stance leg, but not step leg, irrespective of post-visual preview delay. Confidence bands display plus-or-minus one standard error for the mean (SEM) and the simple slopes (b’s) are provided. * *p* < 0.05.

Post-visual preview delay had neither a moderating nor main effect χ^2^(8) = 1.47, *p* = 0.993. Although the interaction between leg and SSRT was not significant in the MLM, χ^2^(1) = 3.26, *p* = 0.071, it was retained (Table 1) because of its moderate effect, partial-R^2^ = 0.039. Subsequent simple slopes analyses confirmed the association between muscle activation ratio and SSRT in the stance leg, b = 0.0066, *p* = 0.015, but not the step leg, b = 0.0023, *p* = 0.338 (Figure 3). Post hoc association was strong between muscle activation ratio and SSRT measured on the stance leg, r = 0.606, *p* = 0.024, but not step leg, r = 0.184, *p* = 0.539. 

## 4. Discussion

Our previous study in younger adults revealed a relationship between SSRT and compensatory stepping using an approach similar to the one outlined above. This suggests an individual’s capacity to inhibit a prepotent finger response on a cognitive task is linked to their ability to make a corrective balance response when step suppression is required. In the current study, we explored if this same relationship persists in older and midlife adults. Our results showed that this link is indeed present, but solely in the stance limb. 

The lack of relationship between response suppression in the step leg and the SSRT was unexpected given our recent results in young adults, whereas stance limb results are consistent. It is not entirely clear why this would change with age; it could reflect a strategic shift by older adults to rely more on their stance leg to perform this particular task. An emphasis on the stance leg in the older adults may indicate a stalling tactic to accrue information before committing to a step. This is consistent with Thelen and colleagues’ [37] observation of faster liftoff times in the step leg using a standard lean & release task where no step inhibition was required (e.g., 315ms in their results versus presently reported liftoff times of 471ms). If indeed there is a strategic shift by older adults to purposely delay a step, this may be best captured via plantar flexor EMG in a stance limb since these muscles directly act to resist a forward fall. This means our current approach using an ankle dorsiflexor (TA) to represent the step decision could miss some aspects of the postural response. Similarly, a general stiffening response (i.e., co-contraction) at the ankle joint would go undetected using TA alone. Co-contraction is a common finding in older adults performing postural tasks [38,39,40], and this may compromise response flexibility [41,42]. For example, co-contraction may impose a challenge to the motor system if excessive antagonist activation at the ankle joint must be first overcome to allow step initiation. Also, a default stiffening response would obscure detecting a distinct TA signal leading to a step. Thus, any co-contraction at the ankle joint could theoretically mask a relationship between stopping capacity measured by the SSRT and the leg response, if one does exist. To resolve this, future work will need to record from a wider variety of leg muscles.

It is further unclear what specific role tibialis anterior has in the stance limb during this task. Admittedly, the original rationale for focusing on recordings from tibialis anterior was to capture foot liftoff during a forward step [23]. However, it is notable that this muscle’s actions are not limited to ankle dorsiflexion—for example, it serves a stabilizing role during midstance in gait [43]. In a similar way, tibialis anterior activity in the stance limb during this task may help stabilize the body while executing a forward step. Any decision on whether to step or not likely arises in the stance leg first as it needs to accept body weight before allowing lift of the opposing leg. This may also explain why the relationship between SSRT and the stance leg response emerges as early as 200 ms.

The concept of an age-linked decline of inhibitory function was first advanced by Hasher and Zacks [44]. However, it is important to note that much of this decline may be attributed to general processing speed delays rather than impaired response inhibition [45]. In fact, when proportional slowing due to age is factored in, recent meta-analyses have shown that a specific age-related inhibitory control deficit is much less uniform than once thought (i.e., the age-effect varies across multiple forms of inhibitory control) [46,47]. A recent meta-analysis assessed the evidence for a uniform decline in inhibitory control with age [46] and found older adults exhibited impaired ability to suppress dominant responses relative to young adults, but showed a preserved ability to ignore distracting information and response interference (e.g., Flanker, Stroop, and Simon tasks). Thus, tasks that emphasize the suppression of dominant responses, such as the SST, exhibit age-related decline. What this means is that, given how different tests reflect unique aspects of inhibitory control, each with varying susceptibility to age [46], they may not equally relate to falls. A possible implication of these results is that tests that stress action cancellation (e.g., SST) may be particularly informative of the link between cognitive decline and falls in older adults. The SSRT in the current sample of midlife and older adults showed only a modest decrement when compared with our previous results in young adults [23] (193 ms vs. 175 ms). However, this was also associated with a slower go reaction time (501 ms vs. 424 ms), which may suggest a strategic tradeoff between speed and accuracy. It is interesting to note that such minor changes in seated cognitive test results coincided with altered step performance. Again, this may reflect a more generally cautious approach before acting, but further work is needed to test such speculation. 

In addition to the limitations previously discussed regarding our particular approach [23], we acknowledge some additional limitations. First is a potential order effect, since the SST always preceded the reactive balance task. The original rationale for this order was to avoid physical activity’s influence on subsequent cognitive performance, as single bouts of aerobic [48] or resistance exercise [49] have been shown to alter cognitive performance. However, recent work has shown that inhibitory control can be trained, and that this learning can generalize to untrained inhibitory tasks [50]. This suggests that our participants may have been primed for improved inhibitory capacity in the balance task by first completing the SST. Consequently, our findings should be interpreted with this in mind, and future studies should use a randomized task order to offset the influence of condition order on performance. A second consideration is our use of a reactive balance task that actually represents a type of switching task, versus a strict focus on response inhibition alone. That is, participants switch between two actions—a step or compensatory grasp—to restore balance. We opted for this current approach to emphasize ecological validity since complete omission of a response would rarely be useful to avoid a fall. It is possible that a cognitive task which emphasizes switching (e.g., Wisconsin Card Sorting Task [51,52]) may be even more informative on the link between a specific cognitive ability and reactive balance performance. However, it is also important to recognize that the ability to switch between tasks/sets (i.e., cognitive flexibility) relies critically on inhibitory control as a foundation [53]. As a third limitation, we realize that exclusive focus on tibialis anterior may not fully expose how stopping capacity is expressed in this task. Thelen and colleagues [37] measured muscle activity during rapid stepping responses in a lean-and-release task and showed that ankle extensors are heavily engaged in the stance limb leading up to a step. The ability to temporarily delay a forward fall would seem particularly valuable in the present context, where steps must occasionally be suppressed. As mentioned above, such braking could act to buy time and allow a more informed step decision. This is a distinct task demand from the standard lean-and-release, where a rapid forward step proceeds without the need for restraint. Future work measuring activity across several leg muscles, along with ground reaction forces and kinematics, would offer a more comprehensive account of how people accomplish this task. Recording from additional muscles in future research is warranted, given that the limited selection of muscles in the present study offer only a limited representation of the postural response. Predictably, our observed power was relatively low (46%) due to the small sample size. Consequently, the present study does not allow any definitive claims on the relationship between SST performance and step suppression during balance recovery. Future studies will need to investigate this phenomenon using a larger sample (see supplemental material) and explore differences across midlife and older adults.

## 5. Conclusions

Regardless of whether stopping capacity is expressed through a step or stance leg, it appears that the inhibitory control measured by the stop signal task also governs the suppression of compensatory balance reactions where automatic stepping must occasionally be suppressed. Direct evidence that failure to suppress an inappropriate step plays a significant factor in the prevalence of falls remains elusive (e.g., real-world video capture). However, the link between falls in community-dwelling seniors and performance on reactive stepping tasks—particularly choice stepping reaction tasks where response inhibition is required [17]—provides compelling indirect support for such a mechanism. Beyond the theoretical value in understanding that a common mechanism serves inhibitory control, these findings illustrated that a simple seated test of response inhibition could be used clinically to provide insight into a specific cognitive risk factor related to the increased frequency of falls as we age.

## Figures and Tables

**Table 1:** Parameter Estimates of a Two-Level Random Intercepts Multilevel Model (MLM-RI) for muscle response ratio (Mean EMG_300ms_ Reach/Step), where leg (stance or step) moderates the association with the Stop Signal Reaction Time (SSRT, grand mean centered at 193.2 ms). Notes: This single MLM utilized six aggregated muscle response ratios per unique combination of the two legs (stance and step) and three post-visual preview delays (200 ms, 400 ms, 600 ms) per participant: n_t_ = 60 ratios (level one units) nested within 10 participants (level 2 units). Additionally, the estimated slope (b) values are very small due to the units:muscle response ratio per one millisecond (ms) in SSRT. Wald t-tests for estimated slope significance test use Satterthwaite’s method for degrees of freedom and are provided despite significance being assessed by comparing nested models via the likelihood ratio tests (LRTs). The model’s marginal pseudo-R^2^ = 0.239.

Fixed Effects	Estimated Slope b (SE)	Walde Significancee *p*-Value	Marginal Partiale Pseudo-R^2^
Intercept	0.9220 (0.064)	<0.001 **	
Main Effects			
SSRT, ms	0.0023 (0.002)	0.338	0.024
Leg, Stance vs. Step	−0.1710 (0.064)	0.010 *	0.083
Interaction			
SSRT × Leg	−0.0043 (0.002)	0.078 ^†^	0.039
Random Effects	Variance	*p*-value	
Participants Intercepts	0.0202	0.021 *	
Residual Error	0.0627		

^†^*p* < 0.10, * *p* < 0.05, ** *p* < 0.001, ms = millisecond, units for SSRT are ms.

## Data Availability

The data presented in this study are openly available in the Open Science Framework at https://osf.io/2j3fe/files/ (accessed on 19 January 2021).
